# Thrombocytopenia in Systemic Lupus Erythematosus: Clinical Manifestations, Treatment, and Prognosis in 230 Patients

**DOI:** 10.1097/MD.0000000000002818

**Published:** 2016-02-12

**Authors:** Jin-Hee Jung, Moon-Seung Soh, Young-Hwan Ahn, Yoo-Jin Um, Ju-Yang Jung, Chang-Hee Suh, Hyoun-Ah Kim

**Affiliations:** From the Department of Rheumatology, Ajou University School of Medicine, Suwon, South Korea.

## Abstract

The aim of the study was to examine the clinical characteristics and prognosis according to severity of thrombocytopenia and response to treatment for thrombocytopenia in patients with systemic lupus erythematosus (SLE).

We retrospectively evaluated 230 SLE patients with thrombocytopenia, and reviewed their clinical data and laboratory findings. Thrombocytopenia was defined as platelet counts under 100,000/mm^3^, and patients were divided into 3 thrombocytopenia groups according to severity: mild (platelet counts >50,000/mm^3^), moderate (>20,000/mm^3^, ≤50,000/mm^3^), and severe (≤20,000/mm^3^). Clinical characteristics, treatments, and prognoses were compared among the groups. Furthermore, complete remission of thrombocytopenia was defined as platelet counts >100,000/mm^3^ after treatment.

There was no significant difference in clinical or laboratory findings among the groups according to severity of thrombocytopenia. However, hemorrhagic complications were more frequent in severe thrombocytopenia (*P* < 0.001) and mortality was also higher (*P* = 0.001). Complete remission was achieved in 85.2% of patients. The clinical characteristics and modality of treatment did not differ between the patients with and without complete remission. Mortality in patients with complete remission (1.5%) was significantly lower than in those without complete remission (29.4%, *P* < 0.001). Survival was significantly higher in patients with complete remission from thrombocytopenia (odds ratio = 0.049, 95% confidence interval: 0.013–0.191, *P* < 0.001).

The severity of thrombocytopenia in SLE patients can be a useful independent prognostic factor to predict survival. Moreover, complete remission of thrombocytopenia after treatment is an important prognostic factor. The severity of thrombocytopenia and response to treatment should be closely monitored to predict prognosis in SLE patients.

## INTRODUCTION

Systemic lupus erythematosus (SLE) is an autoimmune disease affecting diverse organs of the body and causing chronic inflammation. It shows a broad spectrum of clinical manifestations and is associated with several autoantibodies.^1^ Hematologic abnormalities, including thrombocytopenia and leucopenia, are common clinical manifestations of SLE. Thrombocytopenia is known as one of the hematological criteria of SLE, according to the American College of Rheumatology (ACR) classification criteria.^2^ Its prevalence has been estimated to range from 10% to 40%, but severe thrombocytopenia is relatively uncommon.^3,4^

There have been several studies about the association between thrombocytopenia and prognosis in SLE patients.^4–9^ In previous studies, thrombocytopenia has been shown to be associated with other severe clinical manifestations of SLE, such as neuropsychiatric symptoms, kidney involvement, and hemolytic anemia.^5,8–10^ It is also known to have an association with the prognosis of SLE, including death.^5,8,9,11–13^ However, the characteristics and prognosis according to severity of thrombocytopenia have been examined in few studies. Moreover, the prognosis according to remission of thrombocytopenia is unknown.

Therefore, in this study, we retrospectively reviewed patients with SLE who developed thrombocytopenia, and analyzed differences in clinical and laboratory findings and prognosis according to severity of thrombocytopenia. Furthermore, we investigated the treatment of thrombocytopenia, and whether remission of thrombocytopenia was associated with other clinical manifestations and prognosis.

## METHODS

### Subjects

We retrospectively reviewed 267 SLE patients with thrombocytopenia who attended the Department of Rheumatology of Ajou University Hospital from July 1997 to May 2015. All patients met 4 or more of the ACR criteria for the diagnosis of SLE, and all patients had antinuclear antibodies (ANA).^2^ Thrombocytopenia secondary to lupus was defined according to the ACR criteria as a platelet count of <100,000/mm^3^. Thirty-seven patients were excluded with other causes related to thrombocytpenia, such as drug-induced thrombocytpenia, sepsis, chemotherapy for combined malignancy, hematological disease (diagnosed by bone marrow biopsy), and liver cirrhosis. As a result, 230 patients were enrolled.

We divided patients according to severity of thrombocytopenia into 3 groups (mild, moderate, and severe), and compared baseline demographic, clinical, and laboratory findings. Mild thrombocytopenia was defined as platelet counts of >50,000/mm^3^, whereas platelet counts between 20,000/mm^3^ and 50,000/mm^3^ were classified as moderate thrombocytopenia, and severe thrombocytopenia was defined as platelet counts of ≤20,000/mm^3^.

Patients were excluded if their medical records were incomplete or insufficient for diagnosis of SLE at the time of diagnosis and throughout the follow-up period. This study was approved by the Institutional Review Board of our hospital (AJIRB-MED-OBS-15-233).

### Variables

We ascertained the age at the time the lowest thrombocytopenia appeared, gender, and smoking history of the patients. We reviewed laboratory findings and clinical manifestations at the time that the platelet count was lowest and throughout the follow-up period. Laboratory results including complete blood counts, C-reactive protein, erythrocyte sedimentation rate, complement (C3 and C4), and autoantibodies were recorded. Autoantibodies included ANA, anti-double-stranded DNA (anti-dsDNA) antibody, anti-Sm, anti-RNP, anti-Ro, anti-La, IgG and IgM anticardiolipin antibody, and lupus anticoagulant. We also investigated clinical manifestations, such as mucocutaneous, musculoskeletal, pulmonary, cardiac, renal, neurological, and hematological manifestations described by the ACR. Disease activity at the time of thrombocytopenia was assessed using the SLE Disease Activity Index (SLEDAI).^14^

We also reviewed medications used for managing thrombocytopenia. The use of glucocorticoids, hydroxychloroquine, cyclophosphamide, azathioprine, tacrolimus, danazol, intravenous immunoglobulin, and rituximab was recorded. In addition, the initial dose of glucocorticoids (prednisone equivalent) for controlling thrombocytopenia was calculated.

We investigated numbers of hospitalization and chief complaints at that time of the patients throughout the follow-up period. A relapse of thrombocytopenia was defined as a reduction of the platelet count <100,000/mm^3^ after complete remission (at least 2 consecutive increases of platelet counts >100,000/mm^3^). We counted number of hospitalizations due to hemorrhagic manifestations, infectious complications, or flares of SLE, defined as an increase of the SLEDAI score of >4 in comparison with the previous SLEDAI score. Follow-up period was defined as the duration from the time of the most severe thrombocytopenia to May 2015 or to the time of follow-up loss, including deaths.

### Statistical Analysis

All data are expressed as means ± standard deviation, and a *P* value of <0.05 was considered to indicate statistical significance. Clinical features and laboratory findings were compared among the groups (mild, moderate, and severe thrombocytopenia) using one-way ANOVA for continuous variables and Pearson χ^2^ test for categorical variables. The characteristics of patients with and without complete remission were compared with independent *t* tests for continuous variables and Pearson χ^2^ for categorical variables. In addition, the variables that had effects on mortality were analyzed by logistic regression analysis. The association between mortality and complete remission of thrombocytopenia was examined with a Cox proportional hazard model. The Kaplan-Meier method was used to prepare survival curves. The SPSS for Windows software (ver. 12.0; SPSS Inc, Chicago, IL) was used for statistical analyses.

## RESULTS

### Clinical Findings in SLE Patients With Thrombocytopenia

Table 1 shows the clinical characteristics and laboratory findings according to severity of thrombocytopenia in 230 SLE patients with thrombocytopenia. Of the 230 patients, 126 (54.8%), 57 (24.8%), and 47 (20.4%) had mild (platelet count >50,000/mm^3^), moderate (platelet count >20,000/mm^3^, ≤50,000/mm^3^), and severe thrombocytopenia (platelet count ≤20,000/mm^3^), respectively. The mean age of the 230 SLE patients with thrombocytopenia was 41.8 ± 15.3 years and 84.3% were women. The mean duration of follow-up was 65.8 ± 48.2 months. Thrombocytopenia developed a mean duration of 23.7 ± 58.1 months after SLE diagnosis. There were no significant differences in mean age or gender distribution among the groups. Clinical features also showed no difference among groups except hemolytic anemia. Hemolytic anemia was more common in patients with moderate (28.1%) and severe thrombocytopenia (27.7%) than in those with mild thrombocytopenia (11.1%, *P* = 0.005). Disease activity did not differ among the groups.

**TABLE 1:**
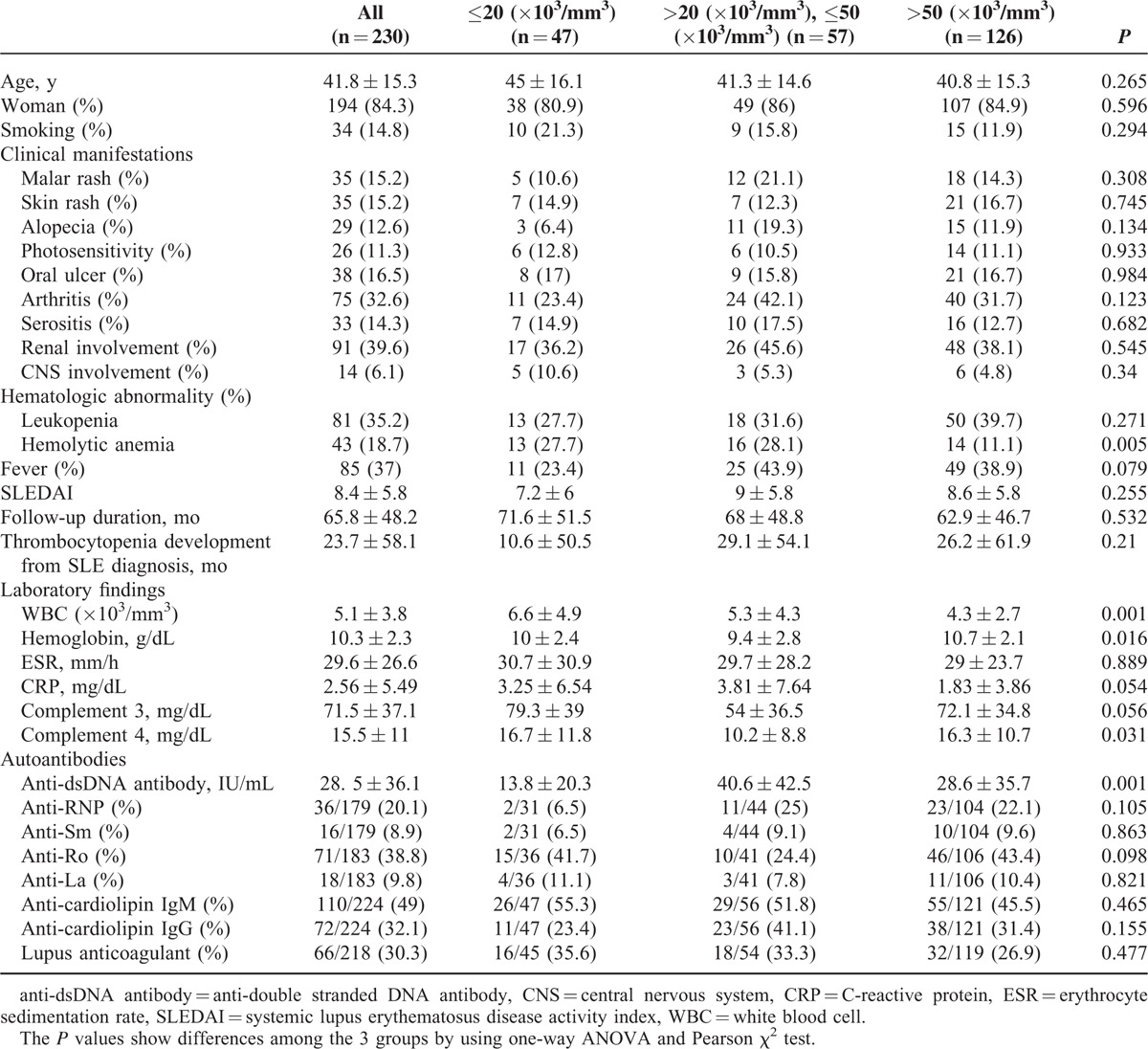
Basal Characteristics and Laboratory Findings of 230 Patients Classified by Severity of Thrombocytopenia

White blood cell count and hemoglobin levels were significantly different among the groups. Hypocomplementemia 4 were most common in the moderate thrombocytopenia group (*P* = 0.031), and the mean titer of anti-dsDNA was highest in that group (*P* = 0.001).

There was no difference in the use of glucocorticoids or hydroxychloroquine among the groups (Table 2). However, the dose of glucocorticoids used for the initial treatment of thrombocytopenia was higher in the severe thrombocytopenia group (478.4 ± 585.3 mg/d of prednisolone equivalent) versus the mild and moderate thrombocytopenia groups (*P* = 0.001). The use of danazol, azathioprine, cyclophosphamide, intravenous immunoglobulin, and rituximab was more common in severe thrombocytopenia (*P* < 0.001, 0.033, <0.001, <0.001, and 0.02, respectively). Three patients had splenectomies for the treatment of thrombocytopenia; all of them had severe thrombocytopenia.

**TABLE 2:**
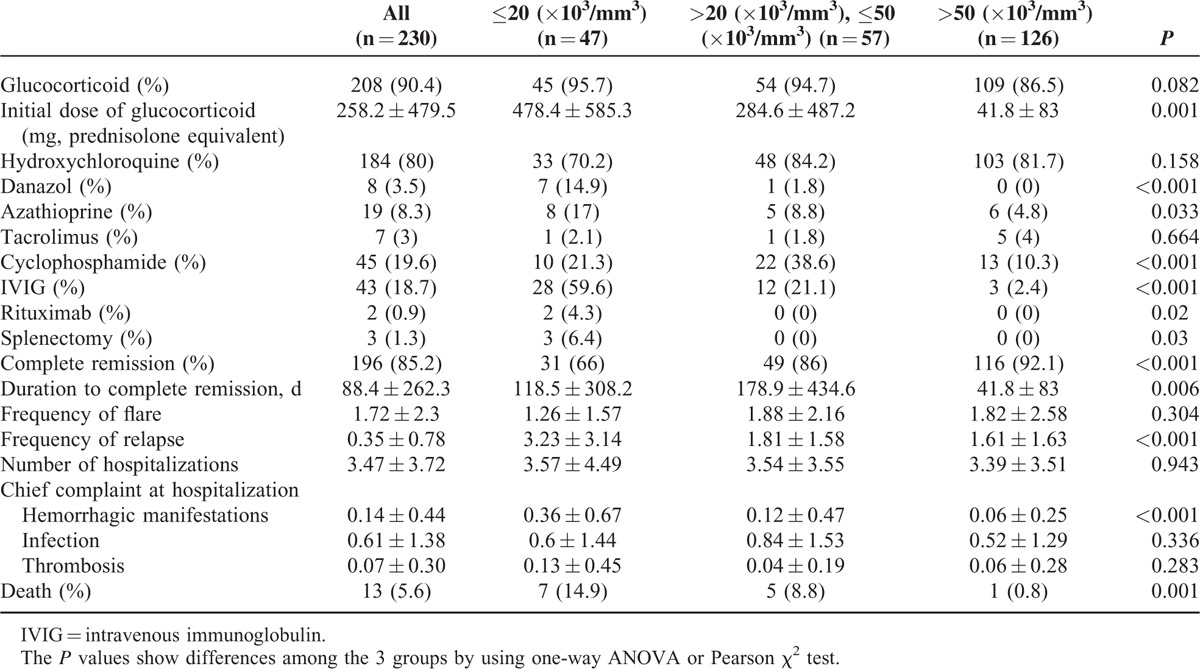
Treatment and Prognosis of 230 Patients Classified by Severity of Thrombocytopenia

In patients with severe thrombocytopenia, the rate of complete remission was lower (31 patients, 66%) than in those with mild (116 patients, 92.1%) or moderate thrombocytopenia (49 patients, 86%, *P* < 0.001). Total numbers of hospitalizations were not different among the groups. However, relapse was more frequent in patients with severe thrombocytopenia (3.23 ± 3.14) than in patients with mild (1.61 ± 1.63) or moderate (1.81 ± 1.58, *P* < 0.001) thrombocytopenia. Hospitalizations due to hemorrhagic manifestations were more frequent in the severe thrombocytopenia group (0.36 ± 0.67, *P* < 0.001). Mortality was significantly elevated according to the severity of thrombocytopenia (14.9% vs 8.8% vs 0.8%, *P* = 0.001; Table 2).

### Comparison Between Patients With and Without Complete Remission

We divided the patients into 2 groups according to achieving for complete remission of thrombocytopenia after treatment. In total, 196 (85.2%) patients achieved complete remission, defined as a platelet count >100,000/mm^3^ in 2 consecutive tests after treatment. The mean age differed significantly; 40.1 ± 14.5 years in patients with complete remission and 51.1 ± 16.7 years in those without complete remission (*P* < 0.001). Other clinical features of SLE showed no difference between the 2 groups. The mean SLEDAI score was 8.7 ± 5.7 in patients with complete remission compared with 6.7 ± 6.3 in those without complete remission; this difference was not significant. Anti-dsDNA antibody was higher in the complete remission group (*P* < 0.001; Table 3).

**TABLE 3:**
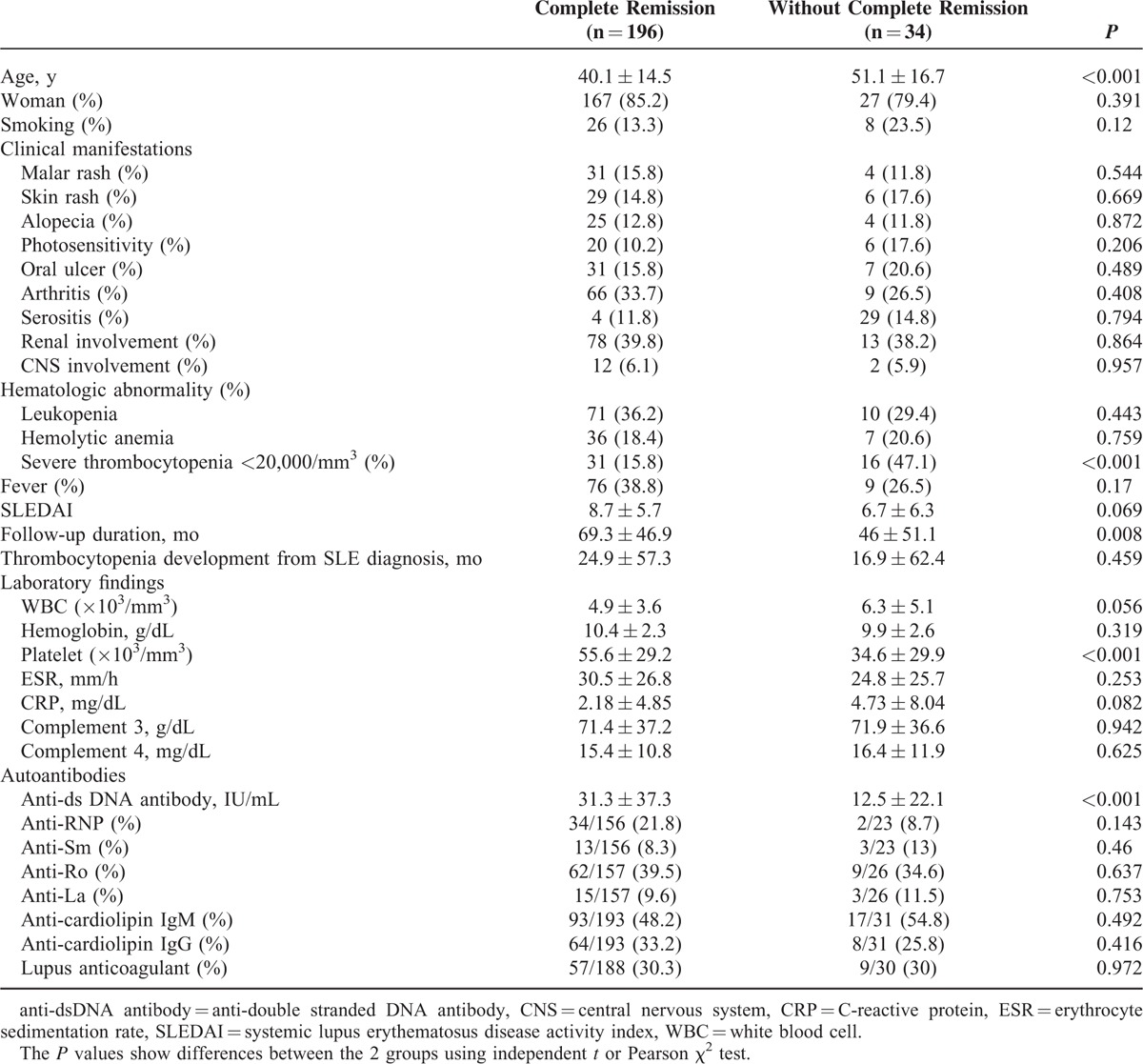
Basal Characteristics and Laboratory Findings According to Complete Remission of Thrombocytopenia

There was no difference in treatment between the 2 groups except use of danazol. The use of danazol was more frequent in the group without complete remission (11.8%, *P* = 0.004; Table 4). The frequency of hospitalization was similar between the 2 groups. Mortality in patients with complete remission (1.5%) was significantly lower than in those without complete remission (29.4%, *P* < 0.001).

**TABLE 4:**
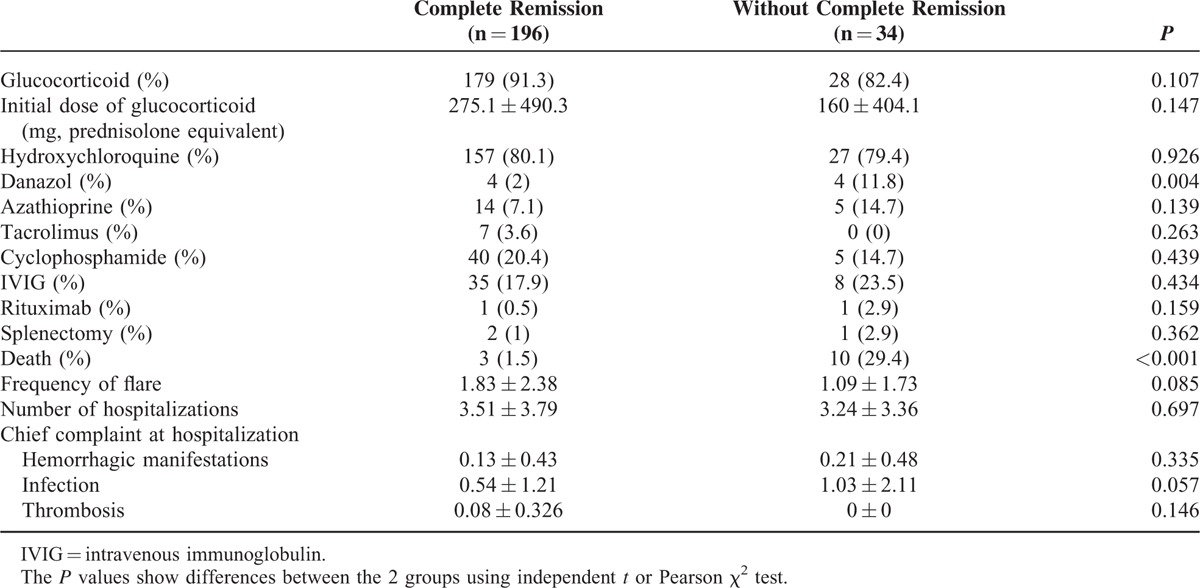
Treatment and Prognosis According to Complete Remission of Thrombocytopenia

### Variables Affecting Mortality in SLE Patients With Thrombocytopenia

**FIGURE 1:**
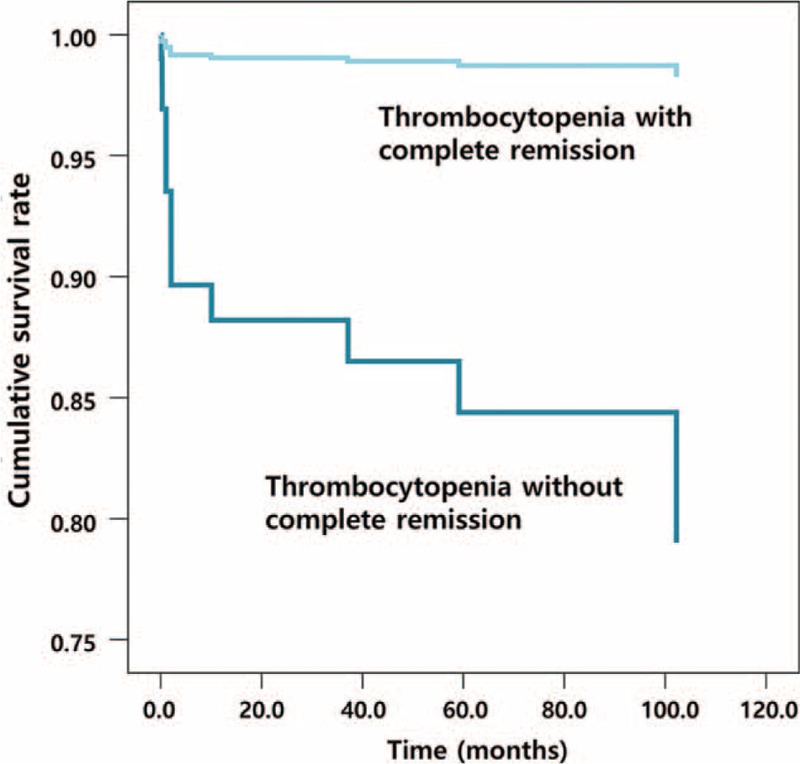
Overall survival rate in patients with systemic lupus erythematosus and thrombocytopenia according to complete remission. Mortality in patients with complete remission was significantly lower than in patients without complete remission (OR = 0.049, 95% CI = 0.013–0.191, *P* < 0.001).

We analyzed the variables affecting the mortality (Table 5). Disease activity, presenting as SLEDAI score, was significantly related to mortality (odds ratio [OR] = 1.111, 95% confidence interval [CI]: 1.015–1.216, *P* = 0.022). Mortality was significantly lower in the group with complete remission (OR = 0.049, 95% CI: 0.013–0.191, *P* < 0.001; Figure 1). Furthermore, we compared mortality according to the treatment of thrombocytopenia. We divided patients according to 5 treatment steps: patients used only hydroxychloroquine for step 1, with glucocorticoids regardless of the use of hydroxychloroquine for step 2; patients were treated with glucocorticoids and cyclophosphamide regardless of the use of hydroxychloroquine in step 3, and were treated with glucocorticoids and intravenous immunoglobulin in step 4; finally, patients underwent a splenectomy or used rituximab in step 5. Among these treatment groups, there was no significant difference in mortality rate. This means the complete remission from thrombocytopenia was an independent predictor of survival.

**TABLE 5:**
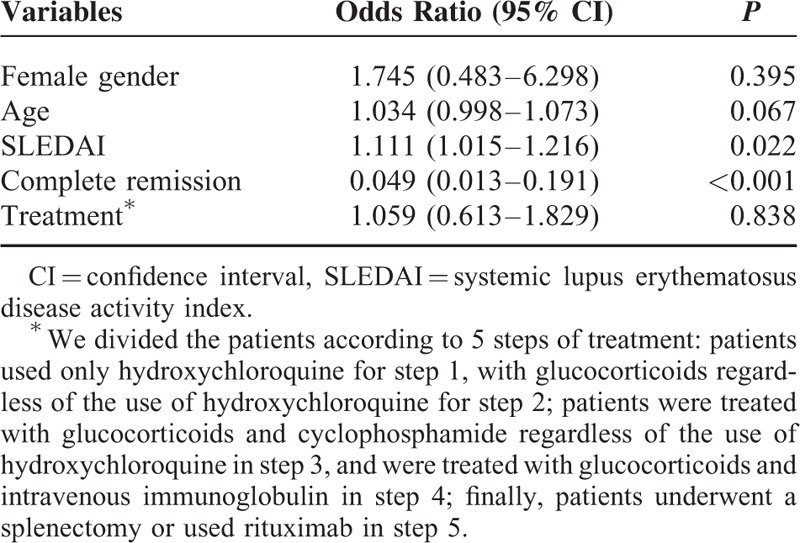
Variables Independently Associated With Mortality in Patients With Systemic Erythematosus Lupus and Thrombocytopenia

The causes of death were variable. Infection, including pneumonia and other sepsis, was most common (7 patients, 53.8%). Hemorrhagic manifestations, like intracranial hemorrhage and hemoptysis, were also causes of death in 2 patients. Two patients died due to renal failure and pulmonary edema caused by disease flare with lupus nephritis. In addition, 2 patients expired because of their malignancies (ovarian cancer and anal cancer).

## DISCUSSION

This is the first reported study to analyze differences in clinical manifestations, treatment, and prognosis according to severity of thrombocytopenia in SLE patients. Furthermore, we evaluated the clinical characteristics of patients who were recovered to platelet counts >100,000/mm^3^, and assessed a possible relationship between complete remission from thrombocytopenia and prognosis.

Thrombocytopenia is a common clinical manifestation in SLE, and several investigators have reported close associations between thrombocytopenia and serious clinical manifestations.^4,13^ In some studies, patients who were thrombocytopenic more frequently exhibited neurological manifestations, kidney disorders, and hematological abnormalities.^5,6,8–10,15,16^ Low complement levels and elevated anti-dsDNA antibody have also been observed among patients with thrombocytopenia.^5,15^ Furthermore, most previous studies showed that disease activity also correlated with thrombocytopenia.^5^ In this study, we evaluated whether several clinical manifestations, and disease activity, of SLE showed correlations with the severity of thrombocytopenia, although we did not compare the SLE patients who were thrombocytopenic to those who were not. Our data showed that the severity of thrombocytopenia was not related to other clinical manifestations, including neurological symptoms, kidney involvement, and disease activity. However, hemolytic anemia was more frequent in patients with platelet counts <50,000/mm^3^ than those with mild thrombocytopenia. In addition, the patients with moderate thrombocytopenia (platelet count >20,000/mm^3^, ≤50,000/mm^3^) had higher anti-dsDNA antibody levels and lower complement levels, and it maybe reflecting a direct effect of SLE disease activity than the patients with mild or severe thrombocytopenia.

Because of the pathogenesis of thrombocytopenia in SLE, which is immune system-mediated, glucocorticoids are used as the first-line treatment.^17^ For patients who fail to respond to glucocorticoids or those who require continuous moderate doses of glucocorticoids to treat the thrombocytopenia, second-line therapeutic agents, including azathioprine, cyclophosphamide, danazol, and intravenous gamma globulin, have been used to treat thrombocytopenia in SLE.^17–19^ There are many case reports indicating that hydroxychloroquine, intermittent cyclophosphamide, danazol, intravenous immunoglobulin, and anti-CD20 antibody (rituximab) had positive effect in treating thrombocytopenia.^18–25^ In this study, second-line therapeutic agents, such as danazol, azathioprine, cyclophosphamide, and intravenous immunoglobulin, were used mainly in patients with severe thrombocytopenia. A recent study reported that 88% of patients responded to treatment, and that a complete response (platelet counts >150,000/mm^3^) was observed in 61%.^26^ Although their definition of complete remission was different, in our study we showed that 85.2% of patients experienced complete remission, with platelet counts >100,000/mm^3^ after treatment. We analyzed the characteristics of the patients with complete remission, and they tended to be younger than those without complete remission and had less severe thrombocytopenia.

Factors associated with prognosis of SLE are known to include man gender, age at first diagnosis of SLE, and renal, heart, and central nervous system involvement at disease onset.^7^ In previous studies, the mortality of thrombocytopenic patients with SLE was significantly high.^5,6,13,15^ The mortality of thrombocytopenic patients was 24%, which was a significantly higher rate compared with those who were not thrombocytopenic (hazard ratio [HR] = 2.855, *P* < 0.001).^5^ A recent study reported a mortality rate in thrombocytopenic patients of 17.1%, which was significantly different to nonthrombocytopenic patients (HR = 1.79, *P* = 0.045).^6^ In our study, the mortality of thrombocytopenic patients was 5.6%, lower than in previous studies. Furthermore, we found that the severity of thrombocytopenia affected the mortality rate. In patients with severe thrombocytopenia, a significantly higher mortality was seen at 14.9%, compared with patients with moderate and mild thrombocytopenia (8.8% and 0.8%, respectively). In previous studies, other clinical outcomes, such as disease flare, infectious complications, and major hemorrhagic events, have also been associated with thrombocytopenia.^6^ In this study, in a comparison according to severity of thrombocytopenia, rates of hospitalization for hemorrhagic complications and relapse of thrombocytopenia were higher in severe thrombocytopenic patients. However, hospitalizations for other causes, including disease flare and infection, did not differ among the groups. Our data demonstrated that the severity of thrombocytopenia also affected the survival of SLE patients regardless of other severe clinical manifestations, abnormal laboratory findings, and disease activity. Interestingly, we found that thrombocytopenia recovering completely was important with regard to survival. Although the characteristics and treatment did not differ between the patients with and without complete remission, mortality was significantly higher in the patients without complete remission. The patients with complete remission were younger; however, age was not associated with survival in a logistic regression analysis. We found additional prognostic factors for SLE: severity of thrombocytopenia and complete remission of thrombocytopenia.

Our study had several limitations. First, it was based on retrospectively collected data; thus, some data were not available in a few patients. For example, severe thrombocytopenia is sometimes related with a combination of factors, such as antiphospholipid antibodies or infections. However, given the nature of the current retrospective study, we could not rule out those things in severe cases. Furthermore, selection bias may exist because all patients were from a single center. Second, we did not compare the data for thrombocytopenia with controls having no thrombocytopenic event. However, in previous studies, there were many results where the patients with thrombocytopenia had differences in clinical findings and prognosis in comparison with control groups. Thus, we focused on the clinical characteristics and laboratory findings of thrombocytopenic patients according to degree of thrombocytopenia severity.

In conclusion, the severity of thrombocytopenia in SLE patients can be a useful independent prognostic factor to predict survival. Moreover, response to treatment of thrombocytopenia is also important because failure to achieve complete remission of thrombocytopenia has a close association with mortality. The severity of thrombocytopenia and the response to treatment should be closely monitored to predict the prognosis in SLE patients.
